# Histological data of axons, astrocytes, and myelin in deep subcortical white matter populations

**DOI:** 10.1016/j.dib.2019.103762

**Published:** 2019-03-06

**Authors:** Santiago Coelho, Jose M. Pozo, Marina Costantini, J. Robin Highley, Meghdoot Mozumder, Julie E. Simpson, Paul G. Ince, Alejandro F. Frangi

**Affiliations:** a Centre for Computational Imaging & Simulation Technologies in Biomedicine (CISTIB) and Leeds Institute for Cardiac and Metabolic Medicine (LICAMM), School of Computing & School of Medicine, University of Leeds, Leeds, UK; b CISTIB, Electronic and Electrical Engineering Department, The University of Sheffield, Sheffield, UK; c Sheffield Institute for Translational Neuroscience (SITraN), The University of Sheffield, Sheffield, UK

## Abstract

This immunohistochemistry dataset contains the main structures in deep subcortical white matter (axons, astrocytes, and myelinated axons) in a representative cohort of an ageing population. A set of samples from 90 subjects of the Cognitive Function and Ageing Study (CFAS) were analysed, stratified into three groups of 30 subjects each, in relation to the presence of age-associated deep subcortical lesions. High-resolution microscopy data enables the extraction of valuable information, such as volume fractions, for the construction and validation of diffusion MRI (dMRI) models. The dataset provided here was used in Coelho et al. [1].

**Table:** Specifications table

Subject area	*NeuroImaging*
More specific subject area	*Computational modelling in neuroimaging*
Type of data	*Images in NanoZoomer Digital Pathology Image [.ndpi] format of histologically stained brain sections. Excel sheet with metadata.*
How data was acquired	*Digital whole slide scanner, Nanozoomer XR (Hamamatsu, Photonics Ltd., Hertfordshire, UK).*
Data format	*Raw colour images*
Experimental factors	*Deep White Matter samples from 90 human subjects divided into Control (N* = *30), Normal Appearing White Matter (N* = *30), and Lesion (N* = *30).*
Experimental features	*High resolution microscopy data was acquired for axons, astrocytes, and myelin from three populations of Deep White Matter samples.*
Data source location	*Sheffield, United Kingdom*
Data accessibility	*Full dataset is available upon registration in MULTI-X repository:* https://multi-x.org/*(`Data’--> `OCEAN histology’ folder)*
Related research article	Coelho, S., Pozo, J. M., Costantini, M., Highley, J. R., Mozumder, M., Simpson, J. E., Ince, P. G., Frangi, A. F., 2018. Local volume fraction distributions of axons, astrocytes, and myelin in deep subcortical white matter, NeuroImage 179, 275–287 [1].

**Table:** 

**Value of the data** • Elderly populations with various degrees of Deep Subcortical Lesions (DSCL) were scanned. • Population representative histology data let us extract specific information useful to develop and validate sensitive and specific dMRI models. • Quantitative assessment and comparison of healthy and diseased tissue allows us to quantify disease-specific changes in deep white matter.

## 1 Data

**Fig. 1:**
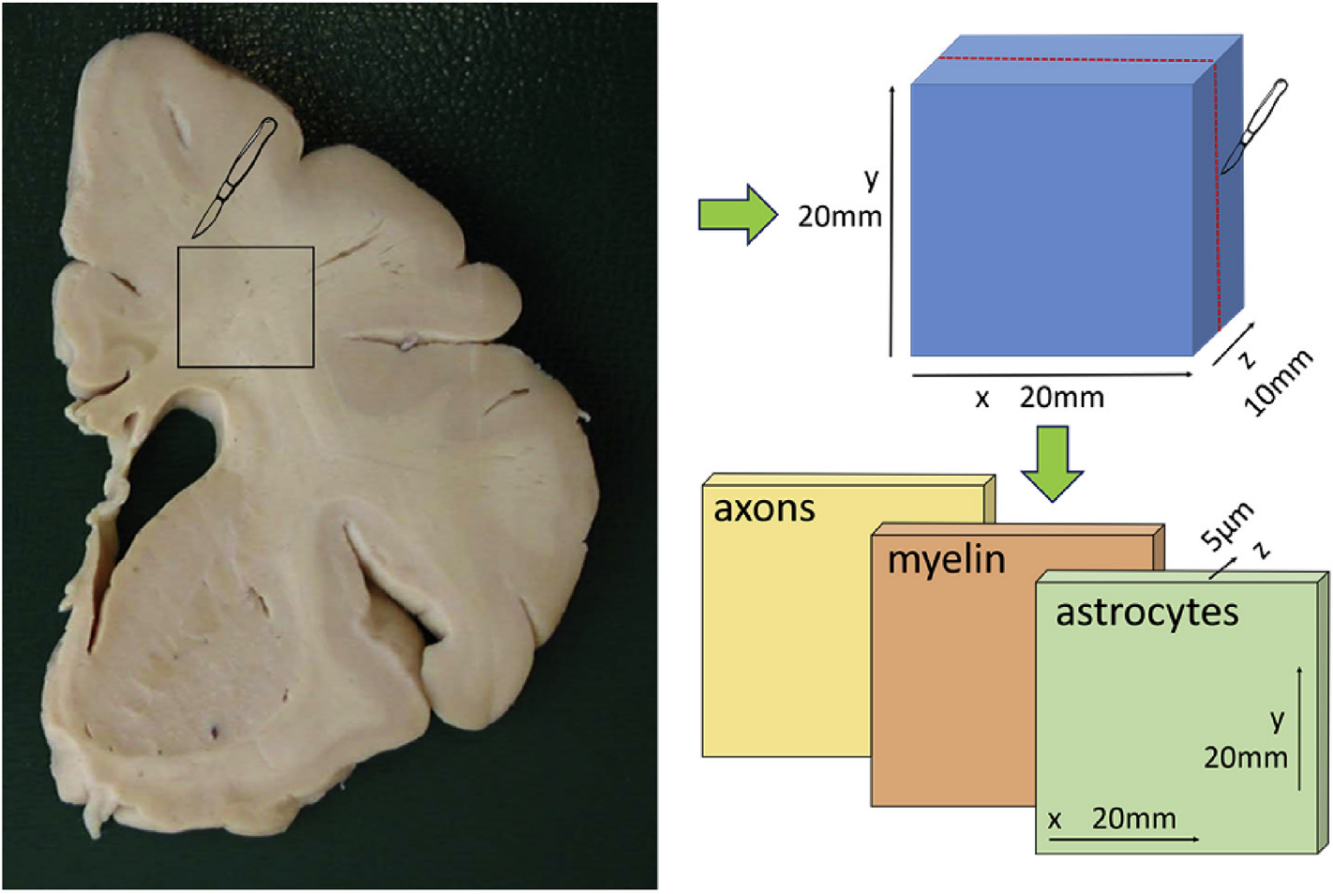
Diagram representing the cuts of the blocks and slices from the coronal sections. Each block belonged to a different subject.

The dataset contains histopathology images of brain deep white matter samples from 90 subjects of the Cognitive Function and Ageing Study (CFAS) cohort. For each sample digital images of axons, astrocytes and myelin markers at 40X magnification are provided (see Fig. 1). The resolution of the images is 0.23 μm/pix. These are available in NanoZoomer Digital Pathology Image (ndpi) format and were allocated in three groups: Control (no DSCLs were present in the subject), Lesion (the sample presented DSCLs), and Normal Appearing White Matter (NAWM, the subject presented DSCLs but not in the sampled tissue).

## 2 Experimental design, materials and methods

Age-associated cerebral white matter lesions can be classified into those within deep white matter of the *centrum semiovale* (deep subcortical lesion, DSCL) and those close to the angles of the lateral ventricles (periventricular lesion, PVL). These are thought to be originated from small vessel-related vascular pathology such as vascular dementia. This work focus on DSCLs, which are associated with loss of myelin components [2] and astrogliosis [3], [4]. To this purpose, several subjects belonging to groups representing healthy and diseased conditions were imaged. Immunohistochemically stained sections of three populations of deep white matter samples were analysed: Control, Lesion, and NAWM.

Samples used in this work came from the Cognitive Function and Ageing Study (CFAS) neuropathology cohort [5], [6]. Brains were removed with the consent of the next of kin and with multicentre research ethics committee approval, according to standard CFAS protocols [7]. Brains were removed within 60 hours of death, one cerebral hemisphere was fixed in buffered formaldehyde and sliced into 10 mm thick coronal slices. These slices were: 1) immediately anterior to the temporal stem (anterior), 2) at the level of the pulvinar (middle), and 3) at the posterior most limit of the occipital horn of the lateral ventricle (posterior). Slices were scanned using T_1_ and T_2_ weighted MRI (details available in [7]). The MR images were rated by three experienced observers (blind to clinical status) and given a score for DSCLs using a modified Scheltens' scale [8]. Following this scoring, the coronal slices were stored in formalin until required for this study (at least four weeks). One block of approximately 20 mm × 20 mm x 10 mm was sampled from one of the slices of every subject. Blocks were allocated in three groups: Control, NAWM, and Lesion. Control blocks were taken from cases where all three levels were scored as 0 on this scale or where only one slice had a score of a maximum of 1. Lesion blocks were taken from regions with a Scheltens' score of 4 or greater. NAWM blocks were taken from lesion free regions of deep white matter in which a DSCL of score 3 or greater was present elsewhere.

The formalin-fixed blocks of tissue were processed to paraffin and embedded in paraffin wax using conventional protocols [7]. Three sections of 5μm thickness were cut from each block for immunohistochemistry (in-plane dimensions of the samples were around 20 mm × 20 mm, see Fig. 1). Sections were collected onto charged slides and underwent Ag retrieval with Access Revelation RTU (A. Menarini Diagnostics Ltd, Winnersh, UK) in a pressure cooker. Sections were immunostained for phosphorylated neurofilament (SMI31, an axonal marker), glial fibrillary acidic protein (GFAP, an astrocyte marker), and proteolipid protein (PLP, a myelin marker) using an intelliPATH FLX system (A. Menarini Diagnostics Ltd, Winnersh, UK). These immunostaining markers were chosen due to being the best option for analysing *ex vivo* samples of these structures [9], [10], [11]. Immunohistochemistry was performed using a standard ABC method, visualised with diaminobenzidine tetrachloride (DAB), and the sections counterstained with haematoxylin. Prepared sections were scanned and digitised at 40X magnification using a Nanozoomer XR (Hamamatsu, Photonics Ltd., Hertfordshire, UK).

## 3 Dataset structure

Three main folders separate all the samples according to different degrees of DSCLs (Control, NAWM, and Lesion groups). Inside each of these, there is a subfolder for each subject, named by the subject id. Inside each subject-specific folder there are three files with the histological images for axons, astrocytes and myelin, respectively, in NanoZoomer Digital Pathology Image (ndpi) format. An ndpi viewer from Hamamatsu (Hamamatsu, Photonics Ltd., Hertfordshire, UK) is freely available at *https://www.hamamatsu.com/eu/en/product/type/U12388-01/index.html*. The metadata with the age and sex of the subjects and position of the samples is provided as a table in an Excel file named ‘Metadata_sorted’ located in the main folder of the dataset.

## 4 Dataset license

This dataset is distributed with the Creative Commons License CC BY-NC-ND 4.0 (Creative Commons Attribution-NonCommercial-NoDerivatives 4.0). Free download and any non-commercial applications are permitted provided that the source is given full credit. Redistribution of any modification or derivative of the dataset is prohibited. Please cite present article if you use the dataset on your research.
